# Definition of a genetic relatedness cutoff to exclude recent transmission of meticillin-resistant *Staphylococcus aureus*: a genomic epidemiology analysis

**DOI:** 10.1016/S2666-5247(20)30149-X

**Published:** 2020-12

**Authors:** Francesc Coll, Kathy E Raven, Gwenan M Knight, Beth Blane, Ewan M Harrison, Danielle Leek, David A Enoch, Nicholas M Brown, Julian Parkhill, Sharon J Peacock

**Affiliations:** a Department of Infection Biology, Faculty of Infectious and Tropical Diseases, London School of Hygiene & Tropical Medicine, London, UK; b Department of Infectious Disease Epidemiology, Faculty of Epidemiology and Population Health, London School of Hygiene & Tropical Medicine, London, UK; c Department of Medicine, University of Cambridge, Cambridge, UK; d Department of Veterinary Medicine, University of Cambridge, Cambridge, UK; e Human Genetics Programme, Wellcome Sanger Institute, Hinxton, UK; f Public Health England, Cambridge, UK; g Public Health England, London, UK

## Abstract

**Background:**

Whole-genome sequencing (WGS) can be used in genomic epidemiology investigations to confirm or refute outbreaks of bacterial pathogens, and to support targeted and efficient infection control interventions. We aimed to define a genetic relatedness cutoff, quantified as a number of single-nucleotide polymorphisms (SNP), for meticillin-resistant *Staphylococcus aureus* (MRSA), above which recent (ie, within 6 months) patient-to-patient transmission could be ruled out.

**Methods:**

We did a retrospective genomic and epidemiological analysis of MRSA data from two prospective observational cohort studies in the UK to establish SNP cutoffs for genetic relatedness, above which recent transmission was unlikely. We used three separate approaches to calculate these thresholds. First, we applied a linear mixed model to estimate the *S aureus* substitution rate and 95th percentile within-host diversity in a cohort in which multiple isolates were sequenced per individual. Second, we applied a simulated transmission model to this same genomic dataset. Finally, in a second cohort, we determined the genetic distance (ie, the number of SNPs) that would capture 95% of epidemiologically linked cases. We applied the three approaches to both whole-genome and core-genome sequences.

**Findings:**

In the linear mixed model, the estimated substitution rate was roughly 5 whole-genome SNPs (wgSNPs) or 3 core-genome SNPs (cgSNPs) per genome per year, and the 95th percentile within-host diversity was 19 wgSNPs or 10 cgSNPs. The combined SNP cutoffs for detection of MRSA transmission within 6 months per this model were thus 24 wgSNPs or 13 cgSNPs. The simulated transmission model suggested that cutoffs of 17 wgSNPs or 12 cgSNPs would detect 95% of MRSA transmission events within the same timeframe. Finally, in the second cohort, cutoffs of 22 wgSNPs or 11 cgSNPs captured 95% of epidemiologically linked cases within 6 months.

**Interpretation:**

On the basis of our results, we propose conservative cutoffs of 25 wgSNPs or 15 cgSNPS above which transmission of MRSA within the previous 6 months can be ruled out. These cutoffs could potentially be used as part of a genomic sequencing approach to the management of outbreaks of MRSA in conjunction with traditional epidemiological techniques.

**Funding:**

UK Department of Health, Wellcome Trust, UK National Institute for Health Research.

## Introduction

The potential benefits of using bacterial-sequencing techniques in routine clinical practice has been well documented in the past 10 years. One of the first applications of this technology in a clinical context was the use of a benchtop sequencer to retrospectively investigate an outbreak of meticillin-resistant *Staphylococcus aureus* (MRSA) in a neonatal intensive-care unit.^1^ The data generated could distinguish MRSA isolates (and patients) involved in the outbreak from those that were not.^1^ A subsequent investigation^2^ of an MRSA outbreak in a special care baby unit showed that standard practice augmented by sequencing was superior to standard practice alone (in terms of identifying cases that would otherwise have been missed), and resulted in an intervention that brought the outbreak to a close. Sequencing can be used to rule out the possibility of an outbreak when MRSA-positive patients are clustered by chance,^3^ thereby reducing unnecessary investigation and interventions. Prospective sequencing can also detect far more outbreaks than standard infection control surveillance: a genomic surveillance study^4^ of MRSA isolated in a clinical microbiology laboratory in the east of England over 12 months identified 173 separate transmission clusters (cluster sizes ranged from two to 44 cases) involving 598 people, none of which were detected by conventional infection control approaches.

Although the translation of bacterial sequencing has clear benefits for effective infection control practice, cost, automation of sample preparation, easy-to-use genome interpretation tools, and lack of standards for genome analyses and interpretation are barriers to the successful adoption of this technology. A major issue is the lack of evidence for a genetic relatedness cutoff between MRSA isolates that is consistent with recent transmission (ie, within the past 6 months). Definition of such a cutoff is essential to enable consistent decisions to be made by personnel who do not have bioinformatics expertise. A genetic relatedness cutoff would also support rapid triaging of routine prospective bacterial sequence data before epidemiological data are available. These sequencing data could thus function as an early-warning system of related bacterial isolates and could lead to highly targeted investigation and intervention to prevent further spread.

**Research in context**

**Evidence before this study**

We searched PubMed with the terms “whole genome sequencing”, “MRSA”, “transmission” AND “hospital” for original research articles published in English up to March 31, 2020, in which whole-genome sequencing had been applied to study transmission of meticillin-resistant *Staphylococcus aureus* (MRSA) in human populations in hospital settings. Of the 35 studies that we identified, WGS was done retrospectively in 17—mostly in the context of suspected outbreaks. Investigators used genetic relatedness thresholds to confirm or refute recent transmission in only five of these studies. Rather, WGS was used to confirm the clonality and small genetic distances expected between isolates of the same outbreak. In 11 of the 18 prospective studies we identified, investigators used relatedness thresholds to exclude recent transmission of MRSA. Two common approaches have been used to define such thresholds: the maximum number of single-nucleotide polymorphisms (SNPs) observed from the same individual, (previously reported values include 10, 40, 43, and 71 SNPs), or the maximum SNP distance between epidemiologically linked cases, with values of 23 and 50 SNPs reported previously (appendix 1 pp 2–3). In the seven prospective studies in which thresholds were not used, authors generally reported the number of SNPs recorded between epidemiologically linked cases.

**Added value of this study**

In this study, we propose a genetic relatedness threshold of 25 whole-genome or 15 core-genome SNPs, above which recent transmission (ie, within the previous 6 months) of MRSA can be ruled out. Our findings were based on the genomes of MRSA isolates from two large patient cohorts, took into account how much genetic diversity would be expected to accumulate over time and be transmitted between individuals, were informed by epidemiological data, and were validated in a third independent dataset. The robustness of our findings was verified using three independent approaches.

**Implications of all the available evidence**

The genetic relatedness thresholds we calculated have a potential role in genomic sequencing approach to management of outbreaks of MRSA, in which they could be used to exclude patients with MRSA who are unlikely to be part of the same outbreak and to focus on those who will require epidemiological follow-up. Our proposed MRSA relatedness cutoffs could also be adopted by academic and clinical laboratories, who are increasingly implementing genomic surveillance. Our approaches to establishing SNP relatedness cutoffs could potentially be applied to calculate equivalent relatedness cutoffs for other bacterial pathogens.

The simplest way to establish genetic relatedness between bacterial isolates is to count the number of individual nucleotides that differ—ie, the number of single-nucleotide polymorphisms (SNPs)—between their whole or core genome sequences. The SNP cutoff approach places two individuals in the same putative transmission cluster if the genetic relatedness of their bacterial isolates is below a pre-defined number of SNPs. In this study, we aimed to define a SNP cutoff between any two MRSA isolates by applying three methods to two large MRSA datasets including clinical evaluations. We also sought to derive a SNP cutoff based on a core genome that could be applied in any setting irrespective of the dominant *S aureus* or MRSA lineages.

## Methods

### Patient cohorts and epidemiological data

We did a retrospective genomic analysis of MRSA data from two prospective observational cohort studies in the UK. Recruitment, MRSA sampling, and selection criteria for whole-genome sequencing (WGS) for the two cohort studies are described in detail in appendix 1 (pp 12–14). The first cohort study^4^ (cohort 1) was done between April 12, 2012, and April 11, 2013, and the second^5^ (cohort 2) was done between Jan 24 and Nov 1, 2018. The aim of both studies was to identify consecutive individuals with MRSA-positive samples processed by the Clinical Microbiology and Public Health Laboratory at the Cambridge University Hospitals NHS Foundation Trust (Cambridge, UK). The studies were approved by the National Research Ethics Service (reference 11/EE/0499), the National Information Governance Board Ethics and Confidentiality Committee (reference ECC 8-05(h)/2011), and the Cambridge University Hospitals NHS Foundation Trust Research and Development Department (reference A092428). The studies were observational and laboratory-based, and all samples were anonymous and collected as part of routine clinical care. Thus the requirement for patient consent was waived.

Epidemiological data (sample date, sampling location, hospital admission and ward movement data, general practitioner registration, and residential postcode) were obtained via the hospital computer system for all patients who tested positive for MRSA. Potential epidemiological links in cohort 2 were investigated up to 6 months before the first sample in a pair, which was defined as an individual and the person whose MRSA isolates most closely matched their own. Ward and community contacts were regarded as strong epidemiological links whereas hospital contact was considered a weak link (on the basis of the findings of our previous study; appendix 1 p 13).^4^ In this study we also used a previously published^6^ independent collection of *S aureus* genomes collected in an intensive-care unit in Brighton, UK .

In cohort 1 and the independent external collection multiple isolates were sequenced for each participant (collected on either the same or different days), whereas in cohort 2 only one isolate per individual was sequenced.

### Microbiology, DNA sequencing, and genomic analyses

A detailed description of MRSA isolation, bacterial DNA extraction, library preparation, sequencing, and genomic analyses is provided in appendix 1 (pp 13–15). Briefly, DNA was extracted, libraries were prepared, and paired-end sequences were obtained on Illumina (San Diego, CA, USA) sequencing machines (HiSeq2000 in cohort 1, MiniSeq used in cohort 2). The bioinformatic pipelines for de-novo assembly, multi-locus sequence typing from de-novo assemblies, short-read mapping, SNP calling, and quality control for genomic data have been previously described for cohort 1.^4^ The same procedures were also applied to cohort 2 and the independent external collection. Whole-genome SNP (wgSNP) distances were calculated by using the entire EMRSA15 reference genome (accession number HE681097; 2·83 Mb) after extraction of mobile genetic elements (2·67 Mb),^7^ whereas core-genome SNP (cgSNP) distances were calculated on the basis of the portion of the reference genome that corresponded to the species core genome.^8^ We used a genetically and geographically diverse collection of 800 *S aureus* isolates^9^ to derive the species core genome, which consisted of 1766 genes and covered 1·76 Mb (62%) of the reference genome. Isolates from all cohorts were also mapped to ST30 strain MRSA252 (accession number BX571856) to test the effect of using a different reference genome in SNP cutoff calculations.^10^, ^11^

### Procedures

**Figure 1:**
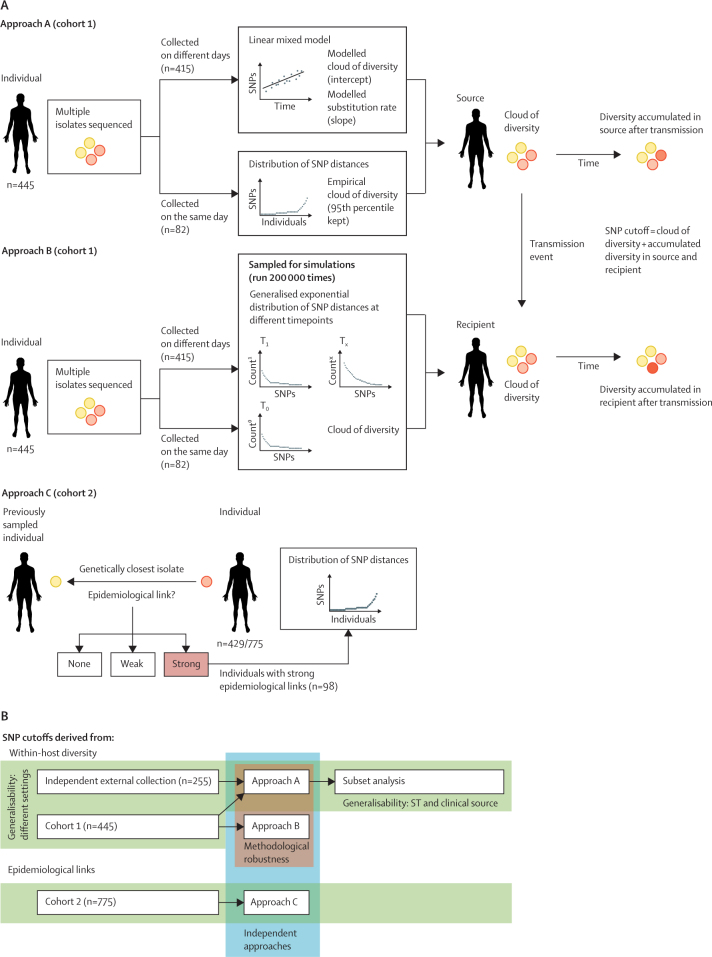
Overview of study design, patient cohorts, and methods (A) Approaches used to calculate a SNP cutoff above which MRSA transmission within a 6-month period is unlikely (appendix 1 pp 15–18). In approach A, a linear mixed model was used to calculate the cloud of diversity and substitution rate for MRSA; these parameters informed the estimation of the SNP cutoff. In approach B, exponential distributions of SNP distances in source and recipient patients were sampled in a simulation model (run 200 000 times) to estimate the SNP cutoff. In approach C, genomic and epidemiological data were integrated to derive the SNP cutoff. (B) Overview and relationships of all approaches and cohorts in this study. MRSA=meticillin-resistant *Staphylococcus aureus*. SNP=single-nucleotide polymorphism. ST=sequence type. T_0_=timespan 0. T_1_=timespan 1. T_x_=generic timespan.

A genetic relatedness cutoff for detection of MRSA transmission was derived by applying three approaches to two different MRSA cohorts. First, we used data from cohort 1 to calculate a theoretical SNP cutoff informed by the *S aureus* substitution rate and within-host diversity (approach A; figure 1A). Second, a simulation model, parameterised with the same data, was used to estimate SNP distances between simulated transmission pairs (approach B; figure 1A). The agreement between these two independent methods in cohort 1 was used to assess their methodological robustness. Third, the distribution of SNP distances among epidemiologically linked cases was derived from cohort 2 (approach C; figure 1A). Finally, we used the independent external collection of genomes to assess the generalisability (ie, in a different geographical location with different *S aureus* strains) of the SNP cutoff (figure 1B).

Before applying approach A, we calculated SNP and time distances between pairs of isolates from the same individual (appendix 2) for cohort 1. Outliers—pairs of isolates representing independent (mixed) strains—were filtered out from further analyses. We used the calculated SNP and time distances to estimate the amount of pre-existing diversity (ie, the cloud of diversity) and the substitution rate. We calculated an empirical cloud of diversity in people from whom more than one MRSA isolate was collected on the same day. For each individual, we calculated the maximum pairwise SNP distance between their isolates, and used the 95th percentile across all individuals. We also applied a linear mixed regression model (LMM) to the data, in which the SNP distance was treated as the dependent variable and time distance as the independent variable (fixed effect). The intercept was interpreted as the amount of pre-existing diversity (which we refer to as modelled cloud of diversity) and was allowed to vary by individual (random effect), as we observed that the empirical cloud of diversity varied by individual (presumably as a result of different duration of colonisation and other factors). We interpreted the slope of the fixed effect as the substitution rate (appendix 1 pp 15–16). Because individuals had a different number of isolate genomes available per sampling date, they were de-duplicated by random subsampling to retain only one isolate per person per date. The reported substitution rates and modelled cloud of diversity are the median values resulting from sub-sampling and running the LMM 100 times. The substitution rate per site per year was calculated for the core genome by dividing the average substitution rate of the core genome by the length of the core genome. The LMM was run with the *lmer* function in the R package *lme4* (version 1.1–23). Plots were created in ggplot2 (version 3.3.1).

In approach A, we assumed that the genetic distance between any two MRSA isolates obtained from different individuals during an outbreak would be dictated by the amount of pre-existing diversity that could be transmitted from the source, plus the number of SNPs that could accumulate over time in the source and recipient hosts. We used the estimated values of the cloud of diversity and substitution rate to derive a SNP cutoff to detect MRSA transmission within 6 months (a frequently used timeframe in infection control investigations) with the following formula: SNP cutoff=(95th percentile of the initial cloud of diversity) + (expected mutation accumulation over 6 months × 2). We used the 95th percentile rather than the maximum cloud of diversity observed to avoid including extreme values at the upper end of the exponential distribution. Expected mutation accumulation over 6 months was doubled to account for the fact that after a transmission event, the MRSA genomes in the source and recipient would accumulate SNPs in different positions in the genome.

The simulation model in approach B was based on data from cohort 1 (appendix 1 pp 16–18). Distributions of SNP distances were generated for source and recipient patients within 6 months of a transmission event. For the source patient, exponential distributions were fitted to the data for within-host SNP distances between isolates sampled on different dates (appendix 1 pp 10–11). From these distributions, we built a generalised exponential distribution of SNP distances by time between sampling to simulate within-host variation between transmission and subsequent time of sampling. For the recipient patient (who was assumed not to be colonised with MRSA at the time of transmission before being colonised by a single strain, so-called transmission bottleneck), the fitted mutation rate from the LMM provided a mean for an exponential distribution of potential SNP distances at any given time. To this SNP distance, we added the SNP distance between the transmitted isolate and the isolates remaining in the source patient, the latter of which was randomly sampled from the distribution of within-host SNP distances between isolates taken on the same day. The simulation model assumed a cohort of 445 transmission pairs (to match cohort 1) and sampled the generalised exponential distributions to give SNP distances from the transmitted isolate in both the source and recipient patients, which were then summed to give a SNP distance. We calculated the SNP distance threshold that would capture 95% of the transmission events. This process was repeated 200 000 times, and the maximum value and range of this threshold over 10 sets of 200 000 runs was used as the simulation model output.

In approach C, we paired each patient in cohort 2 with the individual whose MRSA isolate was the closest genetic match (allowing a maximum genetic distance of 50 SNPs between isolates) and defined the epidemiological contact of each pair as weak, strong, or no contact. If a patient had a strong epidemiological link to the person whose MRSA isolate was the closest genetic match to their own, they were defined as epidemiologically linked cases. Approaches A, B, and C were all implemented in R (version 3.6.3).

We used the external independent dataset of *S aureus* genomes (appendix 3) to assess the generalisability of the SNP cutoff. We derived a SNP cutoff by applying approach A to the external dataset as we did for cohort 1. We then did two subset analyses (also with approach A): one to assess whether the SNP cutoff was affected by the sequence type (ST) of the reference genome used (ST22 or ST30), and another to assess the effect of using *S aureus* isolates from patients with clinically confirmed MRSA infections or from patients who were asymptomatically colonised (because *S aureus* intra-host diversification can differ between infection and colonising sites^12^, ^13^).

### Role of the funding source

The funders of the study had no role in study design, data collection, data analysis, data interpretation, or writing of the Article. All authors had full access to all the data in the study and had final responsibility for the decision to submit for publication.

## Results

Cohort 1 consisted of 1465 people with at least one MRSA-positive sample as reported previously (total sample 2282 MRSA isolates).^4^ In 1006 (69%) people, only one isolate was sequenced. We restricted our analysis to the remaining 459 (31%) people, from whom 1276 MRSA isolates were sequenced (median 2 [range 2–15; IQR 2–3] per patient). The frequency of clonal complexes is detailed in appendix 1 (p 3). Cohort 2 comprised 778 people with at least one MRSA-positive sample. Most patients (n=775 [100%]) provided one MRSA isolate. Overall, 781 isolates were cultured from multisite screens (526 [67%] from typical colonising sites) or diagnostic specimens (255 [33%] from infection sites) and sequenced. In our analysis, we focused on a subset of 429 (55%) individuals who were positive for an MRSA strain that was within 50 wgSNPs of the MRSA carried by at least one other patient, because recent transmission beyond this genetic distance is very unlikely (appendix 1 pp 2–3).^4^

**Figure 2:**
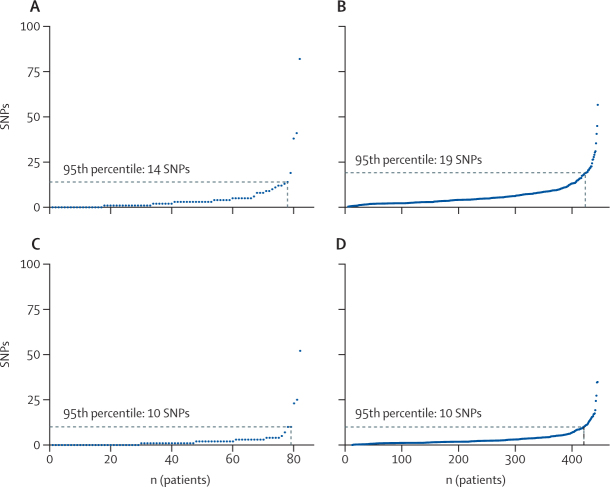
Empirical (A) and modelled (B) clouds of MRSA genetic diversity across individuals in the whole genome and empirical (C) and modelled (D) clouds of MRSA genetic diversity across individuals in the core genome Each datapoint corresponds to the maximum pairwise genetic distance between MRSA isolates from the same individual in cohort 1, measured as the number of SNPs in the whole or genome. The empirical cloud of diversity refers to diversity noted between isolates from the same individual collected on the same day. The modelled cloud of diversity was estimated from a linear mixed model of isolates from the same individual collected on different days. MRSA=meticillin-resistant *Staphylococcus aureus.* SNP=single-nucleotide polymorphism.

Of the 459 patients with multiple MRSA isolates in cohort 1, 14 (3%) were infected with independent (mixed) strains, and a further nine (2%) patients were infected with both the same and independent strains. We removed these outliers and kept the remaining isolate pairwise comparisons from the same patient and strain (1510 isolate pairs from 445 patients; appendix 2). When these data were inputted into the LMM, the average substitution rate per genome per year was 4·70 wgSNPs (95% CI 2·85–6·54) or 2·92 cgSNPs (1·69–4·15). The substitution rate per site per year of the core genome was 1·66 × 10^−6^ (95% CI 9·63 × 10^−7^·–2·36 × 10^−6^), which was similar to that estimated for the whole genome without mobile genetic elements (1·76 × 10^−6^ [95% CI 1·07 × 10^−6^–2·45 × 10^−6^]). Substitution rates were similar irrespective of the reference genome (ST22 or ST30) and portion of the genome used for calling SNPs (whole or core genome; appendix 1 p 4). The 95% CIs for our estimates overlap with *S aureus* substitution rates estimated in other studies (appendix 1 p 4).^14^, ^15^, ^16^, ^17^, ^18^, ^19^, ^20^, ^21^ Both the empirical and modelled cloud of diversity followed the same exponential distribution (figure 2). We chose the 95th percentile value from the modelled distribution (19 wgSNPs or 10 cgSNPs; n=445 patients) rather than that from the empirical distribution (14 wgSNPs or 10 cgSNPs; n=82 individuals) because the former was based on more patients and was thus likely to be more accurate.

In approach A, we added the 95th percentile values from the modelled cloud of diversity distribution (ie, 19 wgSNPs or 10 cgSNPs) to the number of SNPs expected to accumulate over 6 months in the source and recipient hosts (4·70 wgSNPs or 2·92 cgSNPs, rounded to 5 or 3, respectively) to estimate a SNP cutoff for detecting MRSA transmission within the previous 6 months of 24 wgSNPs or 13 cgSNPs (table).

**Table:** SNP cutoffs estimated by different approaches in the whole genome or in the core genome

	SNP cutoff*
**Whole genome**	
Approach A (cohort 1)	24
Approach B (cohort 1)	17 (16–17)†
Approach C (cohort 2)	22
**Core genome**	
Approach A (cohort 1)	13
Approach B (cohort 1)	12 (11–12)†
Approach C (cohort 2)	11

SNP=single-nucleotide polymorphism.

* Within a period of 6 months.

† Maximum (range) over ten sets of 200 000 simulations.

In approach B, according to our simulation model of pairwise MRSA transmission based on the 445 included patients from cohort 1 and run 200 000 times, a maximum of 17 wgSNPs (range 16–17) or 12 cgSNPs (11–12) were needed to capture 95% of transmission events occurring within a period of 6 months.

**Figure 3:**
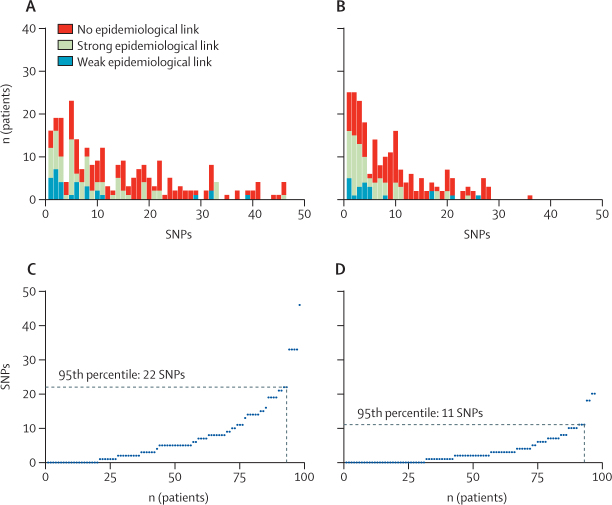
Distribution of SNP distances among epidemiologically linked individuals Number of individuals who had a MRSA isolate genetically linked to that of another individual in cohort 2, grouped by increasing whole-genome (A) or core-genome (B) SNP distances, and colour-coded by the strength of epidemiologically link between them (n=294). (C) and (D) show the subset of patients with strong epidemiological links (n=98). MRSA=meticillin-resistant *Staphylococcus aureus*. SNP=single-nucleotide polymorphism.

In approach C, in which we studied SNP distances among epidemiologically linked cases in cohort 2 (appendix 4), we observed a decline in the number of strong epidemiological links with increasing SNP distances in both the whole and core genome (figure 3A, 3B). Of the 429 individuals who were positive for an MRSA strain with a genetic distance of 50 wgSNPs or less from the MRSA carried by at least one other patient, 294 (69%) had available epidemiological data and 98 (23%) had strong epidemiological links. When we plotted the distribution of SNP distances for these 98 individuals, 22 wgSNPs (figure 3C) or 11 cgSNPs (figure 3D) captured 95% of epidemiologically linked cases. We repeated this analysis for the subset of individuals with strong hospital links (ie, people who, within a 6-month period, had been on the same hospital ward within 1 week of each other) and found that 22 wgSNPs or 7 cgSNPs captured 95% of putative nosocomial transmission (appendix 1 pp 7–8). SNP distances were higher between individuals with community epidemiological links (appendix 1 pp 7–8).

We also derived a SNP cutoff for detection of recent MRSA transmission based on an independent collection of *S aureus* genomes from a different setting,^6^ which mostly consisted of meticillin-susceptible strains and had a different proportions of clonal complexes from those of cohorts 1 and 2 (appendix 1 p 3). 11 167 isolate pairwise comparisons from 255 individuals (appendix 3) were used. The 95th percentile of the empirical cloud of diversity (n=146 individuals) in this dataset was 21 wgSNPs or 11 cgSNPs (appendix 1 p 9) and the annual substitution rate was 4·90 wgSNPs (appendix 1 p 4), which would result in a theoretical SNP cutoff (per approach A) of 26 wgSNPs or 14 cgSNPs—similar to but slightly higher than those calculated based on cohort 1. Our subset analyses showed that the SNP cutoff was unaffected by the sequence type of the reference genome used (appendix 1 p 5) and that SNP cutoffs for isolates collected from infection sites were slightly lower (21 wgSNPs and 11 cgSNPs) than those collected from people asymptomatically colonised with MRSA (25 wgSNPs and 14 cgSNPs; appendix 1 p 6).

## Discussion

Genome sequencing of bacterial pathogens has emerged as the new gold standard technology for the detection of transmission and outbreaks in health-care settings.^6^, ^22^, ^23^ Genetic relatedness thresholds that define the genetic distance expected between recently transmitted bacteria would facilitate the interpretation of genome sequencing data and enable infection control teams to focus their attention on patients with greater probability of being part of an outbreak.^24^ In this genomic epidemiological analysis, we used three different approaches in two patient cohorts asymptomatically colonised or infected with MRSA to establish an SNP cutoff for detection of recent transmission. We calculated cutoffs that ranged from 17 to 24 wgSNPs, or from 11 to 13 cgSNPs. On the basis of these findings, we propose a conservative cutoff of 25 wgSNPs or 15 cgSNPs, above which 95% of recent MRSA transmission events can be ruled out within a period of 6 months.

Cohorts spanning longer periods will be needed to establish SNP cutoffs to detect transmission beyond 6 months. Other cutoffs informed by the maximum genetic distance observed within individuals (eg, 40 SNPs)^6^ or maximum distance between epidemiologically linked cases (eg, 50 SNPs)^4^ have been reported in the literature (appendix 1 pp 2–3) and are likely to capture older transmission events (of several years) that are beyond the timeframe infection prevention and control teams would be investigating. In our study, we noted higher SNP distances between isolates from individuals with community epidemiological links, than between those from individuals with strong hospital epidemiological links. This observation suggests older transmission events (ie, older than 6 months) were being captured, and that a higher SNP cutoff might thus be needed to capture *S aureus* transmission outside hospitals.

We calculated SNP cutoffs for the core genome as well as the whole genome. The advantage of using the core genome is that the chromosome length and content being used to call SNPs will be the same irrespective of the reference genome used and sequence types being compared, and thus the cgSNP cutoff could be used in any setting regardless of the dominant *S aureus* or MRSA lineages. Although we acknowledge that a core-genome approach will never capture as many SNPs as aligning the whole genomes of isolates to each other or to a closer reference genome, our findings suggest that there was sufficient variability in the core genome to capture substitutions rates and within-host diversity, and thus to derive a cgSNP cutoff.

Our methods could be used to define SNP cutoffs for other bacterial pathogens. For more recombinogenic bacteria, as long as genome analysis pipelines can detect and remove recombination, the presence of homologous recombination should not be an obstacle to derivation of similar SNP cutoffs that are based on the accumulation of point mutations.

A SNP cutoff is a simple and intuitive measure of genetic relatedness and could be easily incorporated into automated genome analysis tools and interpreted by non-expert users in a clinical setting. A limitation of this approach is that the likelihood of direct transmission below the cutoff cannot be inferred. The identification of common epidemiological links (such as visits to the same hospital ward, unit, or clinic, shared residential postcodes, or management by the same health-care worker) is still essential to confirm definite transmission. However, the SNP cutoff would reduce the number of patients who require detailed epidemiological follow-up. In cohort 2, for example, use of the 25 wgSNPs cutoff would mean that only 368 (48%) of the 775 MRSA-positive patients would need to be followed up. Savings associated with using our approach compared with universal epidemiological investigation of all MRSA cases might vary depending on local MRSA epidemiology. Another limitation is that SNP cutoffs cannot be used to identify the source and recipient of individual transmission events (directionality), to establish the probability of transmission, or to characterise the overall transmission dynamics of an outbreak. Alternative phylogenetic and probabilistic approaches have been proposed for such questions.^25^, ^26^, ^27^, ^28^

In conclusion, our study provides evidence for a SNP cutoff for MRSA, and describes an approach that can be applied to define SNP cutoffs for other bacterial pathogens as similar large sequencing datasets become available.

## Data sharing

The whole-genome sequences from this study have been deposited at European Nucleotide Archive (study accessions PRJEB3174 and PRJEB32286). Individual accession numbers are listed in appendices 2–4. The R code implementing approach A and B (snp-cutoff.within_host_diversity.R) and approach C (snp-cutoff.between_hosts_links.R) are available on GitHub.
