# Supplementary material for: Definition of a genetic relatedness cutoff to exclude recent transmission of meticillin-resistant Staphylococcus aureus: a genomic epidemiology analysis
Source: Lancet Microbe. 2020 Dec;1(8):e328–35. doi: 10.1016/S2666-5247(20)30149-X (PMC7721685; doi:10.1016/S2666-5247(20)30149-X)
Supplement: Supplementary appendix [file mmc1.pdf]

THE LANCET
Microbe

Supplementary appendix 1

This appendix formed part of the original submission and has been peer reviewed. We post it as supplied by the authors.

Supplement to: Coll F, Raven K E, Knight G M, et al. Definition of a genetic relatedness cutoff to exclude recent transmission of meticillin-resistant *Staphylococcus aureus*: a genomic epidemiology analysis. *Lancet Microbe* 2020; **1:** e328–35.

Appendix 1 for

Defining a genetic relatedness cut-off to exclude recent MRSA transmission in genomic epidemiology investigations

Francesc Coll, Kathy E. Raven, Gwenan M. Knight, Beth Blane, Ewan M. Harrison, Danielle Leek, David A. Enoch, Nicholas M. Brown, Julian Parkhill, Sharon J. Peacock

Table of Contents

Supplementary Table 1 Published studies using WGS to study MRSA transmission in hospitals and SNP cut-offs used (pages 2 and 3)
Supplementary Table 2. Percentage of clonal complexes in each dataset (page 3)
Supplementary Table 3. Effect of using different datasets and reference genomes on the estimation of *S. aureus* substitution rate (page 4)
Supplementary Table 4. MRSA substitution rates estimated in this and other studies (page 4)
Supplementary Table 5. Effect of using different sequence type subsets on the calculation of the SNP cut-off (page 5)
Supplementary Table 6. Effect of using patients with and without *S. aureus* infection on the calculation of the SNP cut-off (page 6)
Supplementary Figure 1. Distribution of SNP distances broken down by type of epidemiological link (pages 7 and 8)
Supplementary Figure 2. Pre-existing S. aureus genetic diversity across individuals in different settings (page 9)
Supplementary Figure 3. Generalised exponential distribution of SNP distances (page 10)
Supplementary Figure 4. Results of the model fit (page 11)
Supplementary Materials and Methods (pages 12 to 18)
References (18 to 21)
Not included in this file:
Appendix 2. Within-host pairwise comparisons of MRSA isolates in cohort 1. (separate excel file)
Appendix 3. Within-host pairwise comparisons of *S. aureus* isolates in the independent collection. (separate excel file)
Appendix 4. Genetic and epidemiological links for MRSA individuals in cohort 2. (separate excel file)

Supplementary Table 1 Published studies using WGS to study MRSA transmission in hospitals and SNP cut-offs used

	Article	PubMed PMID	Study design	Patient transmission investigated?	Setting	SNP cut-off used (SNP distances observed among outbreak isolates*)
1.	Tsujiwaki et al. 2020	32215993	retrospective (no suspected outbreak)	Yes	NICU	<50 SNPs (10 SNPs)
4.	Nurjadi et al. 2020	32112885	prospective	No	NA	NA
6.	Slott et al. 2019	31857121	retrospective (suspected outbreak)	Yes	ICU and OD	Not used (>= 25 SNPs not epidemiologically related cases)
8.	Kristinsdottir et al. 2019	31507231	prospective (suspected outbreak)	Yes	NICU	Not used (0–11 and 0–6 SNP)
13.	Gideskog et al. 2019	30908773	retrospective (suspected outbreak)	Yes	Maternity unit	Not used (7 SNPs)
17.	Cheng et al. 2019	30680562	retrospective (suspected outbreak)	Yes	NICU	Not used (2-17 SNPs)
21.	Manara et al. 2018	30424799	retrospective (no suspected outbreaks)	No	hospital-wide	NA
23.	Kossow et al. 2019	30240815	prospective	Yes	hospital-wide	Not used (4-6 SNPs)
26.	Rubin et al. 2018	30042745	retrospective (suspected outbreak)	Yes	hospital-wide	Not used (50 SNPs, 4-year period)
27.	Earls et al. 2018	30022976	retrospective (suspected outbreak)	Yes	NICU	Not used (1-19 wgMLST allelic differences)
29.	Kossow et al. 2018	29851962	prospective	Yes	hospital-wide	<6 cgMLST allelic differences (no transmission)
31.	Smibert et al. 2018	29501730	prospective	No	ICU	NA
34.	Tosas Auguet et al. 2018	29095965	prospective	Yes	hospital-wide	≤10 SNPs[1]
35.	Toleman et al. 2017<br>Coll et al. 2017	29077854<br>29070701	prospective	Yes	hospital-wide & community	≤50 SNPs[3]
38.	Weterings et al. 2017	28679522	retrospective (suspected outbreak)	Yes	Oncology ward	≤40 SNPs[1] (17 SNPs)
40.	Chow et al. 2017	28475785	prospective	Yes	health-care network	≤60 SNPs[2]
41.	Ankrum et al. 2017	28446577	prospective (only CF patients)	Yes	hospital-wide	≤71 SNPs[1]
43.	Earls et al. 2017	28399151	retrospective (suspected outbreak)	Yes	hospital-wide	≤43 SNPs[1]
45.	Sabat et al. 2017	28106148	retrospective (suspected outbreak)	Yes	hospital-wide	Not used (≤8 SNPs among epidemiologically linked cases)
46.	Azarian et al. 2016	27732618	prospective	Yes	NICU	Not used
47.	Harrison et al. 2016	27716432	prospective	Yes	LTCF	≤40 SNPs[1] & ≤50 SNPs[3]
48.	Mellmann et al. 2016	27558178	prospective	Yes	hospital-wide	≤6 cgMLST allelic differences
49.	Kpeli et al. 2016	27547406	prospective (Buruli ulcer wounds)	Yes	hospital-wide	Not used
52.	Ugolotti et al. 2016	27184087	retrospective (suspected outbreak)	Yes	hospital-wide	>95% of alleles
57.	Kong et al. 2016	26950298	retrospective (suspected outbreak)	Yes	hospital-wide	≤40 SNPs[1]<br>≤23 SNPs[3]<br>(16 SNPs)
61.	Senn et al. 2016	26787833	retrospective (suspected outbreak)	Yes	hospital-wide	Not used, WGS used to confirm clonality
62.	Kinnevey et al. 2016	26582829	prospective	Yes	Acute surgical ward	≤40 SNPs[1]

65.	Bartels et al. 2013	25955776	prospective	Yes	health-care network	Not used (0-14, 59 and 10 SNPs for community clusters; 0-10, and 5 SNPs for ward outbreaks)
66.	Moore et al. 2015	25648940	prospective	Yes	ICU	≤40 SNPs[1]
68.	Tong et al. 2015	25491771	prospective	Yes	ICU	≤60 SNPs[2]
70.	Török et al. 2014	24788657	retrospective (suspected outbreak)	Yes	Haematology ward	Not used (15 SNPs)
73.	Price et al. 2014	24336829	prospective	Yes	ICU	≤40 SNPs[1]
77.	Harris et al. 2013	23158674	retrospective (suspected outbreak)	Yes	SCBU	Not used (20 SNPs)
79.	Köser et al. 2012	22693998	retrospective (suspected outbreak)	Yes	NICU	Not used (41 SNPs)
80.	Eyre et al. 2012	22674929	retrospective (suspected outbreak)	Yes	NICU	Not used (0-3 SNPs)

PubMed studies where whole-genome sequencing was applied to study MRSA transmission in human populations and in a hospital setting (35 out of 80), using the PubMed search terms: "whole genome sequencing", "MRSA", "transmission" AND "hospital". Studies highlighted in grey are the ones where genetic relatedness cut-offs were used. *In backets in the last column, SNP distances observed between outbreak strains and/or epidemiologically linked cases if reported, as either ranges or maximum number of differences observed. [1] SNP cut-offs derived from maximum within-host diversity observed. [2]SNP cut-offs derived from distribution of pairwise SNP distances between patients. [3]SNP cut-offs derived from maximum pairwise distances between epidemiologically linked isolates. Abbreviations: OD, orthopaedics department; ICU, intensive care unit; NICU, neonatal intensive care unit; CF, cystic fibrosis; LTCF, long-term care facility; SCBU, special care baby unit.

Supplementary Table 2. Percentage of clonal complexes in each dataset

Clonal Complex	Cohort 1 % (patient count)	Cohort 2 % (patient count)	Independent collection[1] % (patient count)
1	5.4 (n=79)	5.5 (n=43)	7.9 (n=43)
15	0.6 (n=9)	0.8 (n=6)	8.9 (n=48)
22	70.6 (n=1,035)	48.5 (n=379)	9.4 (n=51)
30	5.5 (n=80)	3.5 (n=27)	19.7 (n=107)
398	0.1 (n=1)	0.8 (n=6)	3.9 (n=21)
45	3.3 (n=49)	11.0 (n=86)	11.4 (n=62)
5	5.4 (n=79)	11.8 (n=92)	8.9 (n=48)
59	2.8 (n=41)	8.7 (n=68)	4.2 (n=23)
8	3.5 (n=51)	4.7 (n=37)	7.0 (n=38)
Other	2.8 (n=41)	4.7 (n=37)	18.6 (n=101)
Total	100 (n=1,465)	100 (n=781)	100 (n=542)

Percentage and counts (shown in brackets) of clonal complexes in each of the three datasets used. Datasets were deduplicated by selecting the earliest available isolate of each individual only. Thus, the total number of individuals per dataset is shown.

Supplementary Table 3. Effect of using different datasets and reference genomes on the estimation of *S. aureus* substitution rate

Dataset	Portion	Reference genome ST	SNPs per genome per year (95 CI)	SNPs per site per year (95 CI)
Cohort 1	Whole genome[1]	ST22	4.70 (2.85 - 6.54)	1.76×10^{-6} (1.07×10^{-6} - 2.45×10^{-6})
		ST30	4.91 (2.55 – 7.27)	1.90×10^{-6} (9.87×10^{-7} - 2.81×10^{-6})
	Core genome[2]	ST22	2.92 (1.69 – 4.15)	1.66×10^{-6} (9.63×10^{-7} - 2.36×10^{-6})
		ST30	2.89 (1.64 – 4.14)	1.65×10^{-6} (9.36×10^{-7} - 2.36×10^{-6})
Price *et al.* 2017	Whole genome[1]	ST22	4.90 (4.24 - 5.57)	1.83×10^{-6} (1.59×10^{-6} - 2.08×10^{-6})
		ST30	6.31 (5.45 - 7.18)	2.44×10^{-6} (2.11×10^{-6} - 2.78×10^{-6})
	Core genome[2]	ST22	2.86 (2.50 – 3.23)	1.63×10^{-6} (1.42×10^{-6} - 1.84×10^{-6})
		ST30	2.79 (2.48 – 3.10)	1.59×10^{-6} (1.42×10^{-6} - 1.77×10^{-6})

S. aureus substitution rates estimated in this study using the whole and core portions of different reference genomes when mapping and calling SNPs. Abbreviations: CI, Confidence Interval; ST: Sequence Type. [1]*Whole chromosome* refers to using the entire reference genome after extracting MGEs to call SNPs. [2]Core genome refers to using the portion of the reference genome that corresponds to the species core-genome.

Supplementary Table 4. *S. aureus* substitution rates estimated in this and other studies

Study	Clonal complex	Substitution rate as substitutions per nucleotide site per year (95% CI)	Substitution rate as number of SNPs per genome per year (95% CI)
This study using cohort 1	Multiple	1.76×10^{-6} (1.07×10^{-6} to 2.45×10^{-6})	4.70 (2.85 - 6.54)
This study using Price *et al.* 2017 data	Multiple	1.83×10^{-6} (1.59×10^{-6} to 2.08×10^{-6})	4.90 (4.24 - 5.57)
Paterson *et al.* 2015 [2]	CC22	8.1×10^{-6} (4.6×10^{-6} to 1.3×10^{-5})	Not reported
Holden *et al.* 2013 [3]	CC22	1.33×10^{-6} (1.2×10^{-6} to 1.4×10^{-6})	Not reported
Uhlemann et al. 2014 [4]	USA300	1.22×10^{-6} (6.04×10^{-7} to 1.86×10^{-6})	~ 3
Tewhey *et al.* 2013 [5]	USA300	1.7×10^{-6} (4.5×10^{-7} to 2.9×10^{-6})	Not reported
Tewhey *et al.* 2013 [5]	USA300 (SSTI isolates)	2.6×10^{-6} (9.1×10^{-7} to 4.3×10^{-6})	Not reported
Baines *et al.* 2015 [6]	ST239	1.6×10^{-6} (1.2×10^{-6} to 2.0×10^{-6})	Not reported
Harris *et al.* 2010 [7]	ST239	3.3×10^{-6} (2.5×10^{-6} to 4.0×10^{-6})	8.67
Nubel et al. 2010 [8]	ST225	2.0×10^{-6} (1.2×10^{-6} to 2.9×10^{-6})	Not reported
McAdam et al. 2012 [9]	CC30	1.42×10^{-6} (1.04×10^{-6} to 1.80×10^{-6})	Not reported

Abbreviations: CC, clonal complex; CI, confidence interval; SSTI, skin and soft-tissue infections.

Supplementary Table 5. Effect of using different sequence type subsets on the calculation of the SNP cut-off

Cohort	Reference genome[1]	Subset (# hosts)[2]	Portion ref. genome[3]	Cloud of diversity (# hosts)[4]	Substitution rate # SNPs per genome per year (95 CI)[5]	Substitution rate per site per year (95 CI)[5]	SNP cut-off (rounded)[6]
Cohort 1	ST22	All (445)	Whole	19.22 (445)	4.70 (2.85 - 6.54)	1.76×10^{-6} (1.07×10^{-6} - 2.45×10^{-6})	23.92 (24)
			Core	10.45 (445)	2.92 (1.69 - 4.15)	1.66×10^{-6} (9.63×10^{-7} - 2.36×10^{-6})	13.37 (13)
		ST22 (340)	Whole	18.99 (340)	5.00 (3.45 - 6.56)	1.87×10^{-6} (1.29×10^{-6} - 2.45×10^{-6})	23.94 (24)
			Core	10.87 (340)	2.96 (1.99 - 3.93)	1.69×10^{-6} (1.13×10^{-6} - 2.24×10^{-6})	13.86 (14)
		Non-ST22 (107)	Whole	20.75 (107)	3.53 (-3.04 - 10.10)	1.32×10^{-6} (-1.19×10^{-6} - 3.78×10^{-6})	24.28 (24)
			Core	10.25 (107)	2.31 (-2.34 - 6.95)	1.31×10^{-6} (-1.33×10^{-6} - 3.96×10^{-6})	12.56 (13)
Independent collection	ST30	All (255)	Whole	21.25 (146)	6.32 (5.45 - 7.18)	2.44×10^{-6} (2.11×10^{-6} - 2.77×10^{-6})	27.57 (28)
			Core	11.50 (146)	2.79 (2.48 - 3.10)	1.59×10^{-6} (1.42×10^{-6} - 1.77×10^{-6})	14.26 (14)
		ST30 (63)	Whole	25.25 (63)	4.24 (3.10 - 5.39)	1.64×10^{-6} (1.20×10^{-6} - 2.08×10^{-6})	29.49 (29)
			Core	9.00 (63)	3.09 (2.68 - 3.49)	1.76×10^{-6} (1.53×10^{-6} - 1.99×10^{-6})	12.09 (12)
		Non-ST30 (197)	Whole	20.20 (113)	6.86 (5.62 - 8.09)	2.65×10^{-6} (2.17×10^{-6} - 3.12×10^{-6})	27.06 (27)
			Core	12.00 (113)	2.79 (2.34 - 3.23)	1.59×10^{-6} (1.34×10^{-6} - 1.85×10^{-6})	14.79 (15)

This table shows the results of a subset analysis comparing substitution rates, cloud of diversity and SNP cut-offs derived using approach A for cohort 1 when using ST22 isolates vs. non-ST22 isolates; and for the independent collection (price2017) when using ST30 vs. non-ST30 isolates. [1]The reference genome was chosen to belong to the most common ST in each cohort. [2]It was not possible to compare ST subsets (e.g. ST22 vs. ST30 isolates) within any cohort due to the small sample sizes of ST subsets except for the major one (ST22 in cohort 1, and ST30 in the independent collection), and thus isolates where split into two groups: major ST vs. all other (minor) STs. [3]Portion of the reference genome used to call SNPs. [4]95 percentile of the modelled cloud of diversity is presented for cohort 1, in place of the empirical one, because the former used many more patients to derive accurate estimates. The 95 percentile of the empirical cloud of diversity is presented for the independent collection. [5]Substitution rates derived from the linear mixed model with 95 confidence intervals (CI). [6]SNP cut-off derived using approach A for a half-a-year period and rounded to the closest integer.

Supplementary Table 6. Effect of using patients with and without *S. aureus* infection on the calculation of the SNP cut-off

Cohort	Reference genome[1]	Subset of patients[2] (# of hosts)	Portion ref. genome[3]	Cloud of diversity (# hosts)[4]	Substitution rate # SNPs per genome per year (95 CI)[5]	Substitution rate per site per year (95 CI)[5]	SNP cut-off (rounded)[6]
Cohort 1	ST22	All (445)	Whole	19.22 (445)	4.70 (2.85 - 6.54)	1.76×10^{-6} (1.07×10^{-6} - 2.45×10^{-6})	23.92 (24)
			Core	10.45 (445)	2.92 (1.69 - 4.15)	1.66×10^{-6} (9.63×10^{-7} - 2.36×10^{-6})	13.37 (13)
		Asymptomatic (201)	Whole	20.10 (201)	5.08 (1.26 - 8.89)	1.90×10^{-6} (4.70×10^{-7} - 3.33×10^{-6})	25.18 (25)
			Core	10.61 (201)	3.33 (0.85 - 5.80)	1.89×10^{-6} (4.84×10^{-7} - 3.30×10^{-6})	13.94 (14)
		With MRSA infection (244)	Whole	17.01 (244)	4.35 (2.18 - 6.52)	2.48×10^{-6} (1.24×10^{-6} - 3.71×10^{-6})	21.36 (21)
			Core	8.81 (244)	2.64 (1.22 - 4.05)	1.50×10^{-6} (6.96×10^{-6} - 2.31×10^{-6})	11.45 (11)
Independent collection	ST22	All (255)	Whole	21.50 (146)	4.91 (4.24 - 5.57)	1.84×10^{-6} (1.59×10^{-6} - 2.08×10^{-6})	26.41 (26)
			Core	10.75 (146)	2.87 (2.50 - 3.24)	1.63×10^{-6} (1.42×10^{-6} - 1.84×10^{-6})	13.62 (14)
		Asymptomatic (221)	Whole	23.00 (119)	4.79 (4.04 - 5.52)	1.79×10^{-6} (1.51×10^{-6} - 2.07×10^{-6})	27.79 (28)
			Core	12.00 (119)	2.82 (2.42 - 3.21)	1.60×10^{-6} (1.38×10^{-6} - 1.83×10^{-6})	14.82 (15)
		With *S. aureus* infection (34)[7]	Whole	9.70 (27)	8.36 (5.02 - 11.69)	3.13×10^{-6} (1.88×10^{-6} - 4.37×10^{-6})	18.06 (18)
			Core	2.00 (27)	3.07 (1.96 - 4.17)	1.75×10^{-6} (1.11×10^{-6} - 2.37×10^{-6})	5.07 (5)

This table shows the results of a subset analysis comparing substitution rates, cloud of diversity and SNP cut-offs derived using approach A when splitting isolates from asymptomatically colonized individuals (using only colonizing isolates) from isolates collected from patients with infection (both colonizing and clinical isolates). [1]The reference genome used to call SNPs. [2]Hosts were classified as symptomatic or asymptomatic based on whether they had a *S. aureus* infection or not, respectively. Both cohort 1 and the independent collection were drawn from prospective studies where clinical specimens had been systematically collected for diagnostic purposes from recruited cases. Thus, individuals were labelled as asymptomatic carriers unless having a clinical specimen collected. [3-6]See footnote in Supplementary Table 5. [7]Note that results obtained for the subset of patients 'With *S. aureus* infection' from the independent collection are regarded as inaccurate due to the low number of individuals (n=34) in this group.

Supplementary Figure 1. Distribution of SNP distances broken down by type of epidemiological link

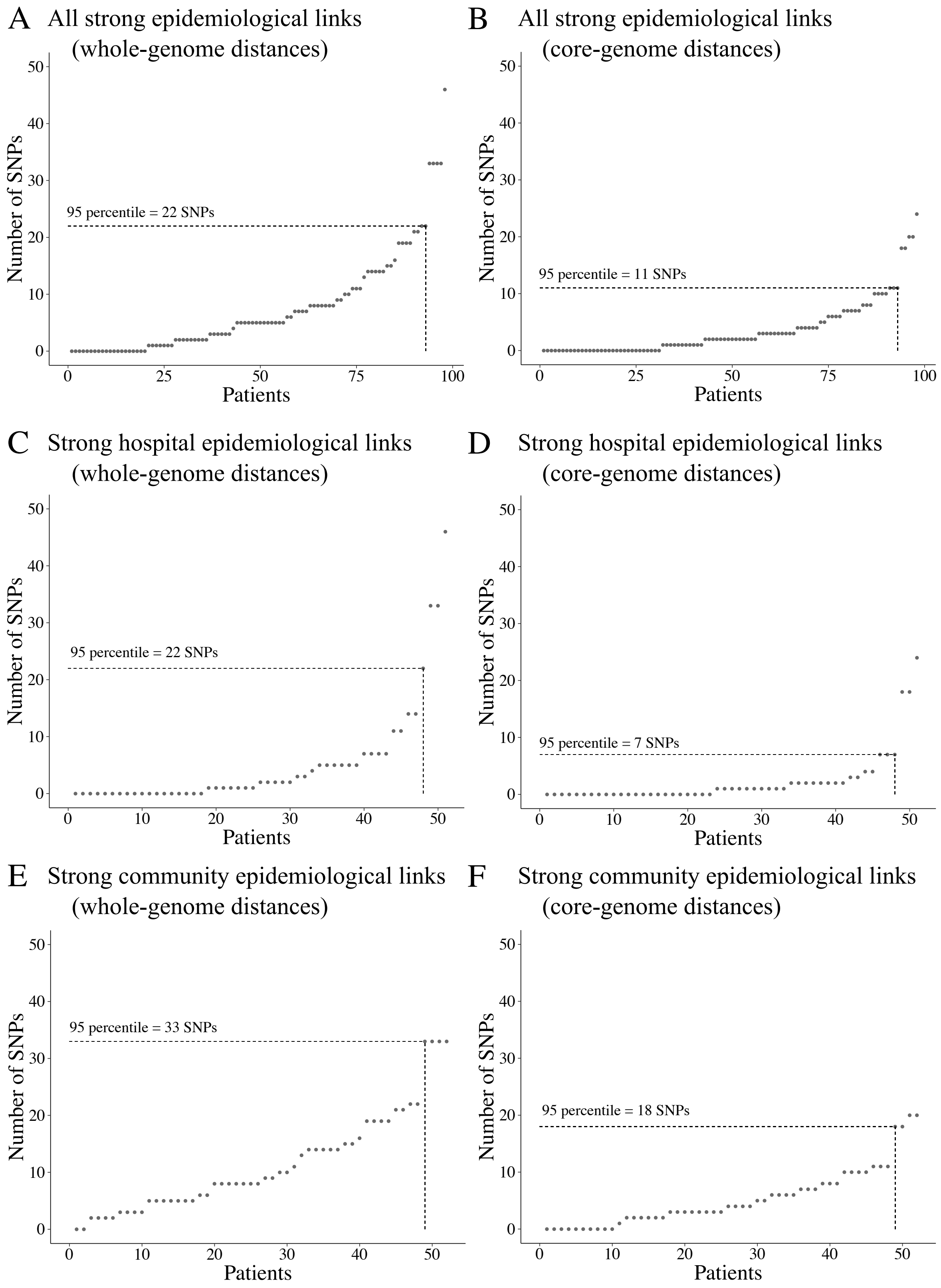

Plots A and B are the same as C and D in the main text Figure 2, which show the distribution of SNP distances among the subset of patients with strong epidemiological links in cohort 2, after excluding pairs of patients with weak or no epidemiological links. Plots C and D here show the distribution of SNP distances among the subset of patients with strong hospital epidemiological links; whereas plots E and F show the distribution among patients with strong community epidemiological links. Both whole-genome (plots A, C and E) and core-genome (B, D and F) SNP distances are shown.

Supplementary Figure 2. Pre-existing *S. aureus* genetic diversity across individuals in different settings

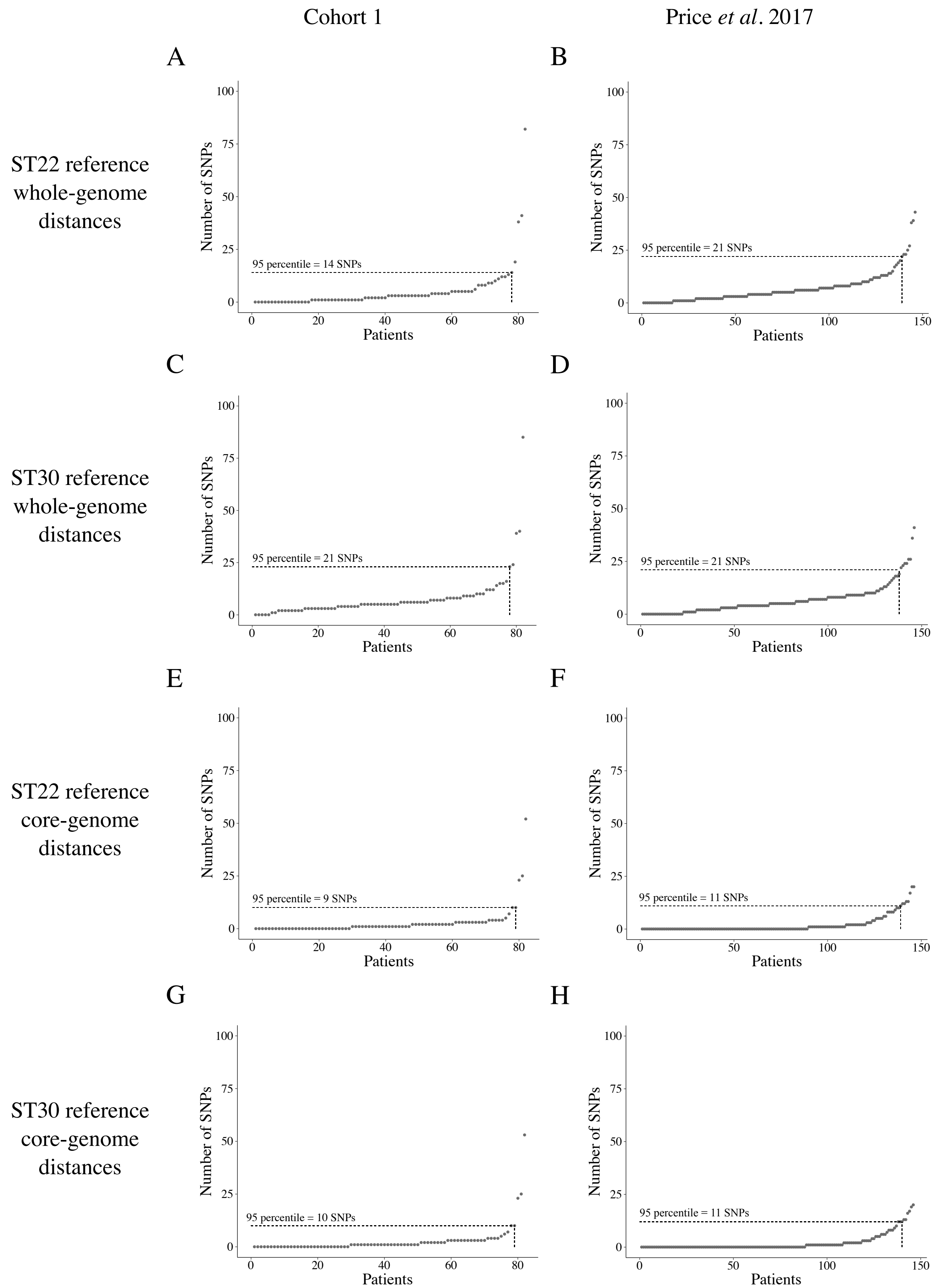

Initial amount of diversity (*empirical cloud of diversity*) calculated from individuals who had multiple *S. aureus* isolates collected on the same day (n=82 in cohort 1 and n=146 in the

independent collection[1]), when using the whole and core-genome portions of two different reference genomes. The reported cloud of diversity (95 percentile) are the median values resulting from randomly sub-sampling each cohort 100 times to retain a single isolate per sampling date.

Supplementary Figure 3. Generalised exponential distribution of SNP distances

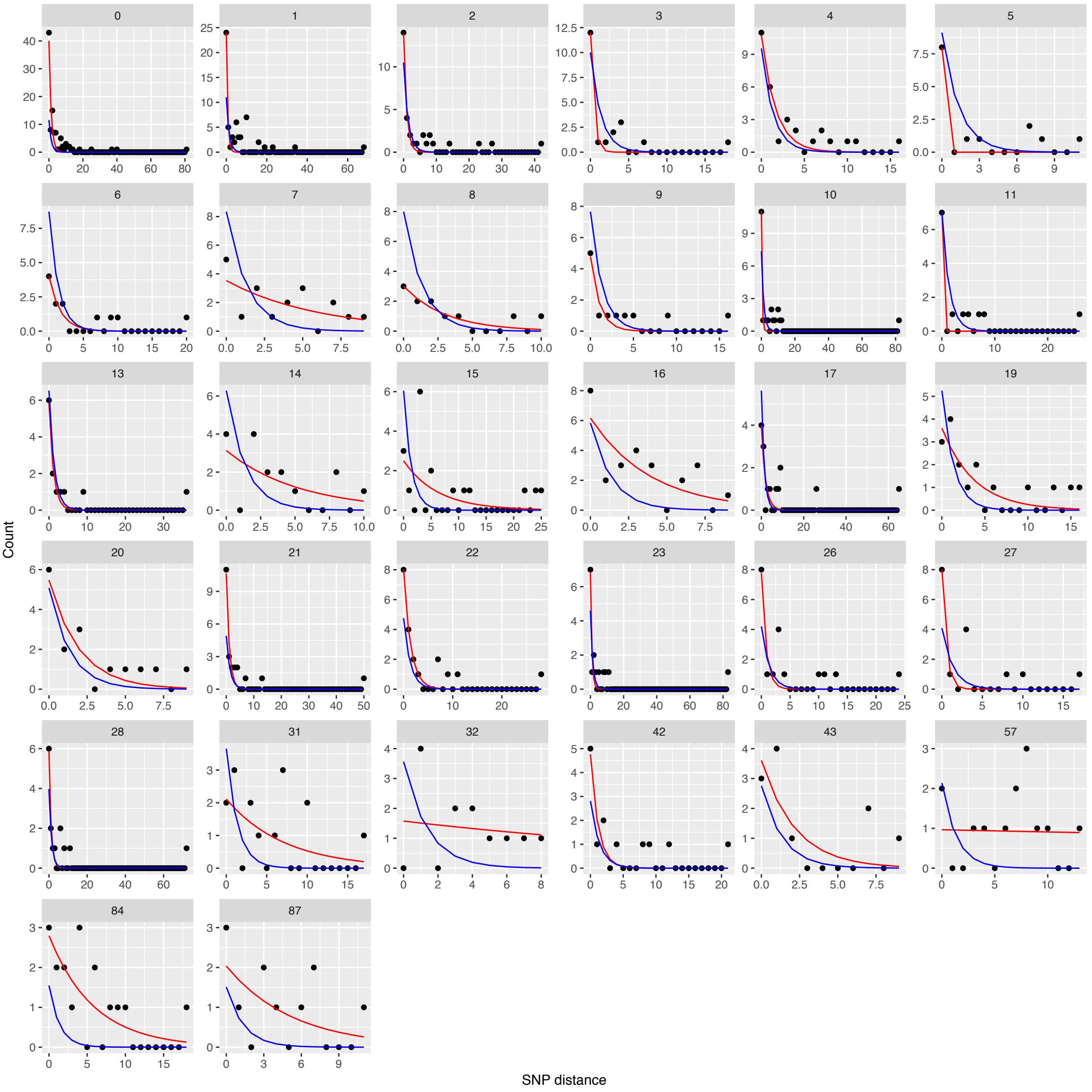

Distribution of SNP distance (x axis) by time between samples (value in days in grey bar) in an individual patient. Red lines are the individual exponential fits, blue is the predicted output from the generalised function.

Supplementary Figure 4. Results of the model fit

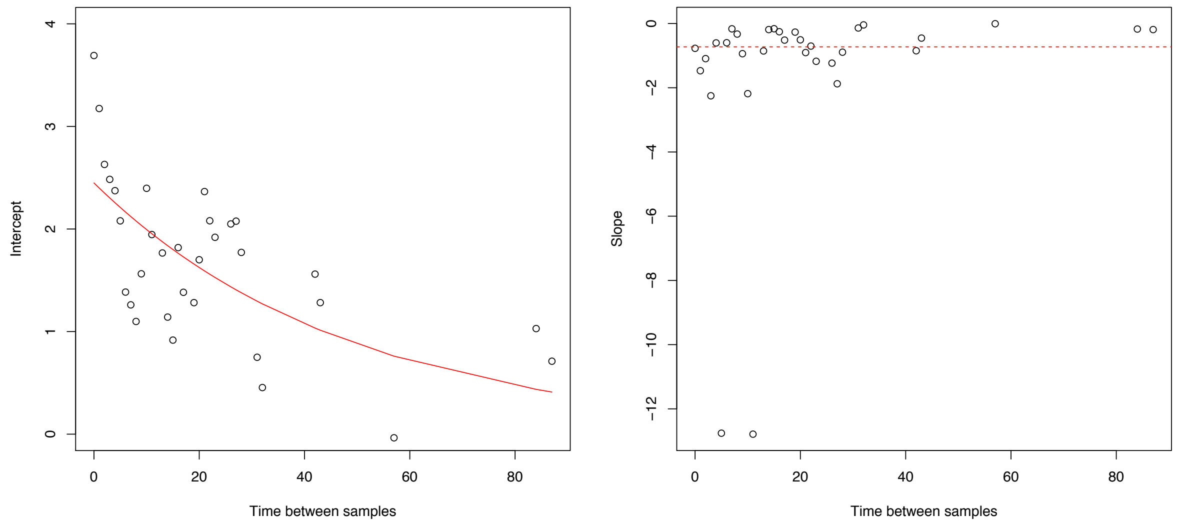

Points are the values for the intercept (left) and slope (right) of the fitted exponential functions for each time between samples with isolates taken from an individual patient. The red lines are the fitted curves to use in the generalised function.

Supplementary Materials and Methods

Details on patient cohorts

Cohort 1 consisted of individuals with MRSA-positive samples obtained from a 12-month prospective observational cohort study[10] conducted between 12 April 2012 and 11 April 2013 to identify consecutive individuals with MRSA-positive samples processed by the Clinical Microbiology and Public Health Laboratory at the Cambridge University Hospitals NHS Foundation Trust. This facility received samples from three hospitals and 75 GP practices in the East of England. All hospital inpatients were routinely screened for MRSA on admission to hospital, and screening was repeated weekly in critical care units. Compliance with mandatory admission screening at the three study hospitals was 85 to 90%. Additional clinical specimens were taken as part of routine clinical care. In the community, there was no formal MRSA screening, and specimens were taken by GPs or community nursing teams for clinical purposes, meaning that coverage was not complete. A total of 2,282 isolates cultured from multisite screens (n=1,619) or diagnostic specimens (n=663) were sequenced. Cohort 2 consisted of individuals with MRSA-positive samples processed by the same microbiology laboratory as cohort 1 between 24 January 2018 and 1 November 2018, wherein the same MRSA screening procedures as in cohort 1 were in place. Consecutive patients with samples that grew MRSA were identified during this period using the hospital IT system (EPIC EMR (Hyperspace® 2014; Epic Systems Corporation)). The first available MRSA isolate from each patient was selected for sequencing, together with all bacteremia isolates. For the first three months these were collected from the freezer archive of the routine laboratory, where available, and for the remaining 6 months the isolates were collected prospectively from routine clinical plates. The first available isolate from each patient was selected, de-duplicating by patient between the 3 month and 6 months collections, together with all bacteremia isolates. A total of

781 isolates cultured from multisite screens (typical colonizing sites, n=526) or diagnostic specimens (taken from infection sites, n=255) were sequenced.

Details on epidemiological definitions

Epidemiological data (sample date, sampling location, hospital admission and ward movement data, GP registration and residential postcode) were obtained for cohort 2 via the hospital computer system (EPIC) for all MRSA-positive cases. For a subset of patients (admitted to two local hospitals), patient movement data could not be retrieved and epidemiological links with other individuals could not be determined, and were labelled as 'Unknown'. Epidemiological links between each pair of MRSA-positive individuals were established through a systematic comparison. *Ward contact* was defined if a case-pair was admitted to the same ward with overlapping dates of admission or within 7 days of each other. *Hospital contact* was defined if cases stayed in hospital at the same time but on different wards. *Community contact* was classified if cases shared a postcode or had their MRSA-positive sample submitted by the same GP practice.

Details on microbiology, DNA sequencing and genomic analysis

For cohort 1, MRSA was isolated by plating screening swabs onto Brilliance MRSA chromogenic medium (Oxoid, Basingstoke, UK) and from all other samples by plating onto Columbia Blood Agar (Oxoid, Basingstoke, UK). *S. aureus* was identified using a commercial latex agglutination kit (Pastorex Staph Plus, Bio Rad Laboratories, Hemel Hempstead, UK). One colony was randomly selected for downstream processing. Bacterial DNA was extracted using the QIAxtractor kit (QIAgen, Hilden, Germany), and anonymised DNA samples were transferred to the Wellcome Sanger Institute. Libraries were prepared with the Nextera DNA Library Preparation kit (Illumina, Cambridge, UK) and sequenced on an Illumina HiSeq 2000.

For cohort 2, freezer archive samples were plated onto Columbia Blood Agar (CBA) using a 1ul loop and incubated overnight at 37°C in air. A single colony was selected for sequencing, and a 10µl loopful was stored at -80°C in Microbank vials (Pro-lab Diagnostics, Bromborough, UK). For prospectively obtained clinical samples, putative *S. aureus* was confirmed using the Staph Latex kit (Pro-lab Diagnostics, Bromborough, UK). A single 2-3mm colony was picked from clinical culture plates using a 1µl loop. Where colonies were smaller than 2mm, several colonies were picked. Where bacterial growth was confluent, a 1µl loopful was taken. If there were several positive plates for one clinical sample, the plate with the least visible background contamination was selected. Isolates were either extracted directly from the clinical plate, or subbed overnight on CBA before processing further. A 10µl loopful was stored at -80°C in Microbank vials. DNA extraction was performed using the QIAgen QIAamp DNA Mini extraction kit (QIAgen, Hilden, Germany) with the amendments described previously.[11] DNA quantification was performed using a Qubit fluorometer (ThermoFisher Scientific, Massachusetts, USA). Sequencing libraries were made using the Illumina Nextera DNA Flex kit (Illumina, Cambridge, UK) with the alterations described previously.[11] Libraries were sequenced on an Illumina MiniSeq with a run time of 13 hours using the high output 150 cycle MiniSeq cartridge and the Generate Fastq workflow.

Isolates from both cohorts were mapped to the same reference genomes: ST22 strain HO 5096 0412 (accession number HE681097) and ST30 strain MRSA252 (accession number BX571856). For each cohort, whole genome alignments were created by replacing called nucleotide alleles along the DNA sequence of the reference genome. Chromosomal regions containing mobile genetic elements (MGEs)[12,13] were removed before calculating pairwise SNP distances from whole genome alignments using pairsnp v0.0.1 (https://github.com/gtonkinhill/pairsnp). SNP distances are counted at positions in the

reference genome shared between any pair of isolates, not at positions present in all isolates (i.e. shared by all isolates). The core genome of *Staphylococcus aureus* was derived from a collection of 800 *S. aureus* from multiple host species [14–16]. Genomes were downloaded from the ENA, *de novo* assembled using *SPAdes* v3.11.0 [17], assemblies improved using the *improve_assembly* pipeline [18] and improved assemblies annotated using *Prokka* v1.14 [19]. The core genome was estimated using *Roary* [20] with default settings. The portion of the reference genome corresponding to the core genome was kept in whole genome alignments to calculate core-genome pairwise SNP distances. To avoid comparing the genomes of divergent strains from the same host, only clonal isolates were kept for further analyses. Clonality was ruled out if isolates belonged to different sequence types (STs) or to the same ST separated by more than 100 wgSNPs. Clonality was ruled in if isolates differed by less than the maximum within-host diversify previously reported (40 wgSNPs). Clonality was investigated for the remaining isolates pairs (differing between 40 to 100 wgSNPs) by making sure both isolates in the pair clustered within the same monophyletic clade in the phylogenetic tree.

Details on linear mixed model (LMM)

It is expected for bacterial strains to diverge over time and, as a result, to accumulate SNP distances proportionally with time at a given rate, known as the substitution rate. As we were interested in testing this in our dataset (cohort 1), as well as in calculating and comparing the substitution rate to those reported elsewhere, we applied a linear regression model to fit SNP distances (dependent variable) as a function of time (independent variable) where the slope of time would correspond to the substitution rate. We kept the slope of the independent variable (substitution rate) constant (fixed effect) as we aim to calculate the average substitution rate across individuals, which is known to be constant in the population. The rationale for using a linear mixed model (LMM) is to allow the intercept (interpreted as the pre-existing amount of

diversity of a MRSA strain at time 0 in an individual, i.e. cloud of diversity) to vary by individual (random effect), as we observed that such pre-exiting diversity is expected to differ by individual, as measured in the subset of subjects with multiple isolate genomes available on the same day.

Before running the LMM on cohort 1 and the independent collection, we de-duplicated these datasets to keep a single isolate per sampling date. This was necessary because while most individuals in cohort 1 and around half of those in the independent collection had a single isolate sequenced per date, some individuals had two or more (81/445 and 146/255, respectively). We hypothesised that having an unequal number of isolates per date could introduce a bias in the LMM estimates. Instead of discarding these individuals, which would have resulted in smaller sample sizes, we chose to keep them by randomly selecting only one isolate per date. The LMM was run 100 times on each random subsample. We chose to repeat this deduplication step and run the LMM on the resulting sub-sample a total of 100 times, but not thousands of times, given the small size of isolate pools to sub-sample from. Specifically, for most individuals in cohort 1, a single isolate was available per sampling date (no deduplication and subsampling needed), and for the rest (n=81), a median of 2 isolates (IQR 2 to 3, maximum 3) were available from which one isolate was randomly selected each time. For the independent collection, we had to deduplicate isolates from 146 individuals, who had a median of 2 isolates (IQR 2 to 2, maximum 74) per sampling date. So, for the vast majority of individuals, 100 de-duplication cycles would capture all their isolates, while retaining a single isolate per sampling date for each LMM analysis. The substitution rates and cloud of diversity reported in the main manuscript are the median values of these 100 runs.

Details on simulation modelling to explore SNP cut-offs for transmission

A simulation model, informed by within-host SNP distributions in cohort 1 (pairwise SNP distances between isolates of the same individual collected at the same or different time points), was built to generate uncertainty ranges around the potential SNP distance between isolates from a (a) *source* and (b) *recipient* individuals. A transmission event was assumed to happen at time t_0 from a source individual (assumed to have an existing distribution of MRSA isolates at t_0) to a recipient individual (assumed not to be colonised by MRSA). An isolate is assumed to be sampled from the source at time t_s after t_0 and from the recipient at time t_r. Here t_s and t_r are assumed to be 6 months, but can be varied in the model.

The SNP distance between the initial transmitted isolate from the source to that at the time of sampling (t_s) was estimated from isolates taken at different times from the same individual. For each time distance in this data, an exponential distribution was fitted to the SNP distributions: $exp(a+bt)$ where t is the time between samples to the SNP distance distribution (Supplementary Figure 3). This utilised the "nls" function (nonlinear least squares) in R. We then plotted the intercept (a) and slope (b) values for each time between samples fit. We found that the best fitting curve for the intercept was another exponential and for the slope, a constant (Supplementary Figure 4). The mean of the slope and the parameters from a fitted exponential distribution for the intercept were used to parameterise a generalised function (red lines in Supplementary Figure 3) that gave the distribution of SNP distances for any sample time distance between isolates from the same host. This was used to sample a SNP distance expected from the transmitted isolate to the sampled isolate taken 6 months later within the source individual.

For the recipient individual, it was assumed that there was a transmission bottleneck, with only one isolate being transferred from the source to the recipient. The variability in the source

patient at time t_0 was sampled from the distribution of SNP distances between multiple isolates taken from one individual on one day (minus those differences present at < 1% of the sample) or the maximum value was used, to capture the variation in potential transmitted isolates.

Once in the new host, this isolate would replicate and form a new population. The average number of mutations in the recipient's population would then be the substitution rate multiplied by the time since transmission (t_r). This mean number of new mutations (sampled from a Poisson distribution) was used as the mean in an exponential distribution of potential SNP distance at 6 months. A sample from this distribution plus the potential transmitted variability gave the SNP distance from the transmission isolate from the "source" patient to the sample taken from the "recipient" 6 months later.

Using the above generated distributions, we sampled SNP distances for a cohort of 445 transmission pairs (the cohort 1 sample size), 200,000 times. For each cohort we calculated the SNP distance threshold that would capture 95% of the transmission events. Due to the stochastic nature of the simulations, the maximum value and range of this threshold over 10 sets of 200,000 simulated cohorts was given as the simulation model output. We explored the variation in the SNP threshold generated by varying the number of simulation runs and found that above 200,000 runs the variation stabilised and a greater number of runs gave little extra information.

References

1 Price JR, Cole K, Bexley A, *et al.* Transmission of Staphylococcus aureus between health-care workers, the environment, and patients in an intensive care unit: a longitudinal cohort study based on whole-genome sequencing. *The Lancet Infectious*

Diseases 2017; **17**: 207–14.

2 Paterson GK, Harrison EM, Murray GGR, *et al.* Capturing the cloud of diversity reveals complexity and heterogeneity of MRSA carriage, infection and transmission. *Nature Communications* 2015; **6**: 6560.

3 Holden MTG, Hsu L-Y, Kurt K, *et al.* A genomic portrait of the emergence, evolution, and global spread of a methicillin-resistant Staphylococcus aureus pandemic. *Genome Research* 2013; **23**: 653–64.

4 Uhlemann A-C, Dordel J, Knox JR, *et al.* Molecular tracing of the emergence, diversification, and transmission of S. aureus sequence type 8 in a New York community. *Proceedings of the National Academy of Sciences* 2014; **111**: 6738–43.

5 Tewhey R, Cannavino CR, Leake J a. D, *et al.* Genetic structure of community acquired methicillin-resistant Staphylococcus aureus USA300. *BMC Genomics* 2012; **13**: 508.

6 Baines SL, Holt KE, Schultz MB, *et al.* Convergent Adaptation in the Dominant Global Hospital Clone ST239 of Methicillin-Resistant Staphylococcus aureus. *mBio* 2015; **6**: e00080-15.

7 Harris SR, Feil EJ, Holden MTG, *et al.* Evolution of MRSA During Hospital Transmission and Intercontinental Spread. *Science* 2010; **327**: 469–74.

8 Nübel U, Dordel J, Kurt K, *et al.* A Timescale for Evolution, Population Expansion, and Spatial Spread of an Emerging Clone of Methicillin-Resistant Staphylococcus aureus. *PLoS Pathogens* 2010; **6**: e1000855.

9 McAdam PR, Templeton KE, Edwards GF, *et al.* Molecular tracing of the emergence, adaptation, and transmission of hospital-associated methicillin-resistant Staphylococcus aureus. *Proceedings of the National Academy of Sciences* 2012; **109**: 9107–12.

10 Coll F, Harrison EM, Toleman MS, *et al.* Longitudinal genomic surveillance of MRSA in the UK reveals transmission patterns in hospitals and the community. *Science Translational Medicine* 2017; **9**: eaak9745.

11 Raven KE, Blane B, Leek D, *et al.* Methodology for Whole-Genome Sequencing of Methicillin-Resistant *Staphylococcus aureus* Isolates in a Routine Hospital Microbiology Laboratory. *Journal of Clinical Microbiology* 2019; **57**. DOI:10.1128/JCM.00180-19.

12 Coll F, Raven K, Reuter S, Holden MTG, Parkhill J, Peacock SJ. Mobile genetic elements on the ST30 strain MRSA252. 2018. DOI:10.6084/m9.figshare.7359272.v2.

13 Coll F, Raven K, Reuter S, Holden MTG, Parkhill J, Peacock SJ. Mobile genetic elements on the ST22 strain HO 5096 0412. 2018. DOI:10.6084/m9.figshare.7059365.v4.

14 Richardson EJ, Bacigalupe R, Harrison EM, *et al.* Gene exchange drives the ecological success of a multi-host bacterial pathogen. *Nature Ecology & Evolution* 2018; published online July 23. DOI:10.1038/s41559-018-0617-0.

15 Coll F, Raven K, Harrison EM, Parkhill J, Peacock SJ. Staphylococcus aureus core genome coordinates on the ST22 strain HO 5096 0412. 2020. DOI:10.6084/m9.figshare.11627193.v2.

16 Coll F, Raven K, Harrison EM, Parkhill J, Peacock SJ. Staphylococcus aureus core genome coordinates on the ST30 strain MRSA252. 2020; published online Jan. DOI:10.6084/m9.figshare.11627226.v1.

17 Bankevich A, Nurk S, Antipov D, *et al.* SPAdes: A New Genome Assembly Algorithm and Its Applications to Single-Cell Sequencing. *Journal of Computational Biology* 2012; **19**: 455–77.

18 Parkhill J, Quail MA, Hunt M, *et al.* Robust high-throughput prokaryote de novo

assembly and improvement pipeline for Illumina data. *Microbial Genomics* 2016; **2**: 1–7.

19 Seemann T. Prokka: rapid prokaryotic genome annotation. *Bioinformatics* 2014; **30**: 2068–9.

20 Page AJ, Cummins CA, Hunt M, *et al.* Roary: rapid large-scale prokaryote pan genome analysis. *Bioinformatics* 2015; **31**: 3691–3.
